# Effect of *Blastocystis* sp. in dengue patients—Increase in the treatment cost and exacerbation of symptoms

**DOI:** 10.1371/journal.pone.0211034

**Published:** 2019-03-20

**Authors:** Gaythri Thergarajan, Suresh Kumar, Subha Bhassu, Sharifah Faridah Binti Syed Omar, Sanjay Rampal

**Affiliations:** 1 Department of Parasitology, Faculty of Medicine, University of Malaya, Kuala Lumpur, Malaysia; 2 Department of Genetics and Molecular Biology, Faculty of Science, University of Malaya, Kuala Lumpur, Malaysia; 3 Department of Medicine, Faculty of Medicine, University of Malaya, Kuala Lumpur, Malaysia; 4 Julius Centre University of Malaya, Department of Social and Preventive Medicine, Faculty of Medicine, University of Malaya, Kuala Lumpur, Malaysia; CEA, FRANCE

## Abstract

Increasing incidences of dengue have become a global health threat with major clinical manifestation including high fever and gastrointestinal symptoms. These symptoms were also expressed among *Blastocystis* sp. infected individuals, a parasite commonly seen in human stools. This parasite has been previously reported to replicate faster upon exposure to high temperature. The present study is a hospitalized-based cross-sectional study involved the collection of faecal sample from dengue patients. Stool examination was done by *in vitro* cultivation to isolate *Blastocystis* sp. Growth pattern of all the positive isolates were analyzed to identify the multiplication rate of *Blastocystis* sp. isolated from dengue patients. Distribution of *Blastocystis* sp. among dengue patients was 23.6%. Dengue patients who were positive for *Blastocystis* sp. infection denoted a significantly higher fever rate reaching 38.73°C (p<0.05) compared to the non-*Blastocystis* sp. infected patients (38.44°C). It was also found that *Blastocystis* sp. infected patients complained of frequenting the toilet more than five times a day (p<0.05) compared to those who were non-*Blastocystis* sp. infected. At the same time, the duration of hospitalization was significantly longer (p<0.05) for *Blastocystis* sp. infected dengue patients compared to the non-*Blastocystis* sp. infected patients. Besides, *Blastocystis* sp. isolated from dengue patients (*in vivo* thermal stress) showed a higher growth rate compared to the non-dengue isolated which was exposed to high temperature (*in vitro* thermal stress). Our findings suggest that presence of *Blastocystis* sp. during dengue infection could trigger the increase of temperature which could be due to highly elevated pro inflammatory cytokines by both parasitic and virus infection. This could justify why the temperature in *Blastocystis* sp. infected dengue patients is higher compared to the non-*Blastocystis* sp. infected patients. Higher temperature could have triggered a greater parasite multiplication rate that contributed to the aggravation of the gastrointestinal symptoms.

## Introduction

*Blastocystis* sp. one of the most widespread and prevalent intestinal parasite in humans has been shown previously to be polymorphic with diverse reproductive processes [1]. The parasite has been distributed widely with prevalence varying between 0.19% and 100% in some cohort groups [2,3]. Sampling populations and differences in the diagnostic approaches could be the contributing reasons for the variation in prevalence [4]. The prevalence of *Blastocystis* sp. infection among the urban and rural communities of Malaysia is 3.40% and 40.7%, respectively [5,6]. The parasite is known to cause bloating stomach, diarrhea, and other gastrointestinal symptoms [7]. Both dengue and *Blastocystis* sp. infection have shown gastrointestinal symptoms but yet till to date there has been no study on the prevalence and correlation of *Blastocystis* sp. infection in dengue patients.

*Blastocystis* sp. has been previously shown to develop survival mechanisms towards various stresses such as the ability to modulate anti-parasitic host nitric oxide (NO) defense by suppressing host epithelial NO production in order to evade nitrosative stress as well as promote colonization in the gut [8]. Besides, oxidative stress also has enhanced the susceptibility and pathogenicity of *Blastocystis* sp. by inhibiting responses of peripheral blood mononuclear cells (PBMC) and immunoglobulin in the host [9]. Apart from these, we previously have shown that this parasite was able to cope with thermal stress by up regulating the heat shock protein 70 (HSP70) genes [10]. Higher parasite growth rate was also seen when sub-cultured after exposing the parasite to a higher temperature. Thus, we postulated that higher temperature induces the proliferation of *Blastocystis* sp. The present study based on the *in vitro* evidence, attempts to assess if parasite isolated from dengue patients having *Blastocystis* sp. would show similar characteristics. In this study, dengue patients have been chosen as an *in vivo* model as they are exposed to high temperature during fever episode.

Dengue is a mosquito-borne viral disease in humans. Dengue has become a major public health problem with social and economic effect. The major clinical manifestation of dengue is fever, ranging from mild fever to potentially fatal dengue shock syndrome together with the presence of gastrointestinal symptoms during fever episode. Diarrhea was found to be the presenting symptoms in almost 50% of patients [11]. These symptoms also commonly presented in other diseases and many reports have emerged from various countries describing the occurrence of co-infections with dengue infections [11].

This study is important as dengue has a global health threat and is hyper-endemic in many countries including Malaysia [12,13]. Since, diarrhea is also the main symptom observed among *Blastocystis* sp. infected individuals, there is an urgent need to identify the distribution of this parasite among patients with dengue infection. Thus, the objectives of this study were to determine the association between *Blastocystis* sp. prevalence and severity of gastrointestinal symptoms in dengue patients and the association between body temperature and *Blastocystis* sp. proliferation.

## Materials and methods

### 1.1. Study population

A hospitalized-based cross-sectional study was conducted on patients who were admitted at University Malaya Medical Centre (UMMC) due to dengue fever. This study was carried out between June 2015 and August 2016 targeting almost 355 potential participants. Each patient was met in person, briefed about the study, provided with a consent form and questions were asked based on a structured questionnaire, but only 89 patients responded by providing their respective faecal sample. The remaining participants could not provide their stool sample due to certain limitations. As there was no baseline study on intestinal parasitic infection among dengue patients, statistical sample size calculation was not carried out. The inclusion criterion of this study was positive diagnosis for dengue non-structural protein-1 (NS1 antigen), a sensitive and timely diagnosis of dengue for early detection of virus infection. The exclusion criterion on the other hand was presence of co-infection along with dengue like *Giardia*, *Entamoeba*, *Dientamoeba*, *Trichuris*, *Ascaris and hookworm* [14–16]. Temperature reading of our respondents throughout the admission period was collected from the electronic patients’ portal of UMMC.

### 1.2. Ethics statement

This study was approved by the Medical Ethics Committee of UMMC (MECID. NO: 20151–984). The study adhered to the tenets of the Helsinki declaration 2013. All human subjects were adults who provided written informed consent.

### 1.3. Sample collection

Stool samples were collected from patients suffering from dengue fever. Each patient was provided with stool cup and sample was processed immediately after collection. Faecal examination was performed by inoculating approximately 50mg of stool sample into 3ml of Jones’ medium supplemented with 10% horse serum [17]. It was incubated at 37°C and examined using light microscope for the subsequent three days [18,19]. A total of 89 stool samples were collected and cultures were continuously examined at 24, 48, and 72 hours.

### 1.4. DNA extraction and polymerase chain reaction

Faecal samples positive for *Blastocystis* sp. using in *vitro* culture method were harvested by centrifugation at 1000g for 5min and washed twice using sterile phosphate buffered saline (PBS) (pH 7.4). Genomic DNA was purified from the harvested parasites using Nucleospin DNA Stool kit (Macherey-Nagel, Germany) according to the manufacturer’s protocol. The concentration and purity of DNA was measured using Nanodrop 2000 (Thermo Scientific, USA). The genomic DNA was amplified using generic primers followed by barcoding of *Blastocystis* sp. [20,21]

### 1.5. Growth pattern analysis

Growth pattern for *Blastocystis* sp. was done based on different thermal conditions. The groups were categorized as *in vitro* and *in vivo*, where the *in vitro* was represented by *Blastocystis* sp. from non-dengue isolates while the *in vivo* was parasite isolated from dengue patients. Parasite culture of the first group was incubated at 37°C (*in vitro* control), the second group was incubated at 41°C for 24 hours and re-cultured at 37°C (*in vitro* thermal stress), and the third group was parasite isolated from dengue patients (*in vivo* thermal stress). The parasites of each isolates were pooled together from day three cultures to make a final concentration of 1×10^5^ cells/ml in 3ml Jones’ medium supplemented with 10% horse serum. A temperature of 41°C was used as it previously showed an increase in parasite count *in vitro* [10]. Moreover 41°C was chosen intentionally as it was a degree more than the usual high fever temperature (40°C) dengue patients often experience during infection [22,23]. Each set was prepared in triplicates and all cultures were kept in airtight tubes. Parasite count was carried out after 24 hours and at every three days interval for up to 13 days. This was done using haemocytometer chamber (Improved Neubauer, Hausser Scientific) with 0.5% Trypan blue solution as viability indicator. Only viable cells which did not take up Trypan blue stain were counted.

### 1.6. Statistical analysis

Statistical analysis was done using chi-square (*χ*^2^*)* for categorical variables, and t-test for continuous variables, a p-value of ≤0.05 were considered as significant. Statistical analysis for growth profile was done using general linear model and a p-value of <0.001 was considered as significant. SPSS version 22 was used in this study.

## Results

Continuous culture examination showed that 21 samples were positive, revealing a distribution of 23.6% *Blastocystis* sp. infection among dengue patients. The genomic DNA of these 21 positive samples were harvested and further subtyped. Sequence analysis categorized positive samples into four subtypes, subtype 1 (7 = 33.33%); subtype 3 (10 = 47.62%); subtype 4 (3 = 14.29%), and subtype 6 (1 = 4.76%). Approximately 14 variables have been analyzed including age, gender, race, lifestyle, having pet, history of dengue among family, symptoms (abdominal pain, diarrhea, flatulence, blood in stool), stool form, frequency of going toilet, day of illness, clinical diagnosis, temperature, whole blood count, and platelet count. However, only the notable ones have been highlighted in this paper. The general information of all the 89 patients who participated in this study has been tabulated in Table 1 based on the presence and absence of *Blastocystis* sp. infection.

**Table 1:** Distribution of *Blastocystis* sp. by various risk factors among dengue patients.

Risk Factors		N	*Blastocystis* sp. Positive	*Blastocystis* sp. Negative	χ^2^	p value
			N (%)	N (%)		
Overall		89	21 (23.6)	68 (76.4)		
Age					0.653	0.419
<35		44	12 (27.3)	32 (72.7)		
≥35		45	9 (20.0)	36 (80.0)		
Gender					4.961	0.026*
Male		49	16 (32.7)	33 (67.3)		
Female		40	5 (12.5)	35 (87.5)		
Clinical diagnosis					0.276	0.600
Dengue fever (DF)		71	12 (16.9)	59 (83.1)		
Dengue haemorrhagic fever (DHF)		18	4 (22.2)	14 (77.8)		
Lifestyle					1.735	0.420
Active, healthy		7	1 (14.3)	6 (85.7)		
Active, busy		50	10 (20.0)	40 (80.0)		
Passive		32	10 (31.3)	22 (68.7)		
Abdominal pain	Yes	31	7 (22.6)	24 (77.4)	0.027	0.869
	No	58	14 (24.1)	44 (75.9)		
Diarrhea	Yes	42	12 (28.6)	30 (71.4)	1.092	0.296
	No	47	9 (19.1)	38 (80.9)		
Stool form					2.527	0.283
Solid		6	0 (0.0)	6 (100.0)		
Soft		49	11 (22.4)	38 (77.6)		
Watery		34	10 (29.4)	24 (70.6)		
Frequency of going toilet					8.282	0.004*
<5 times		79	15 (19.0)	64 (81.0)		
>5 times		10	6 (60.0)	4 (40.00)		
Maximum fever rate (°C)						0.017*
36.0–36.9		7	1 (14.3)	6 (85.7)		
37.0–37.9		32	5 (15.6)	27 (84.4)		
38.0–38.9		32	9 (28.1)	23 (71.9)		
39.0–39.9		5	4 (80.0)	1 (20.0)		

* Represents significance at p<0.05

Chi-square analysis showed that male patients have significantly higher (p<0.05) *Blastocystis* sp. infection compared to female (Table 1). *Blastocystis* sp. infected dengue patients reached a maximum fever rate (39.0–39.9°C) compared to the non-*Blastocystis* sp. infected patients. Those patients also complained of frequenting the toilet more than five times a day (p<0.05) compared to those who were non*-Blastocystis* sp. infected. This concurs with the stool form examination where all *Blastocystis* sp. infected stools were found soft and watery but none was solid. The other factors do not show any presenting effect on the infection rate.

**Fig 1:**
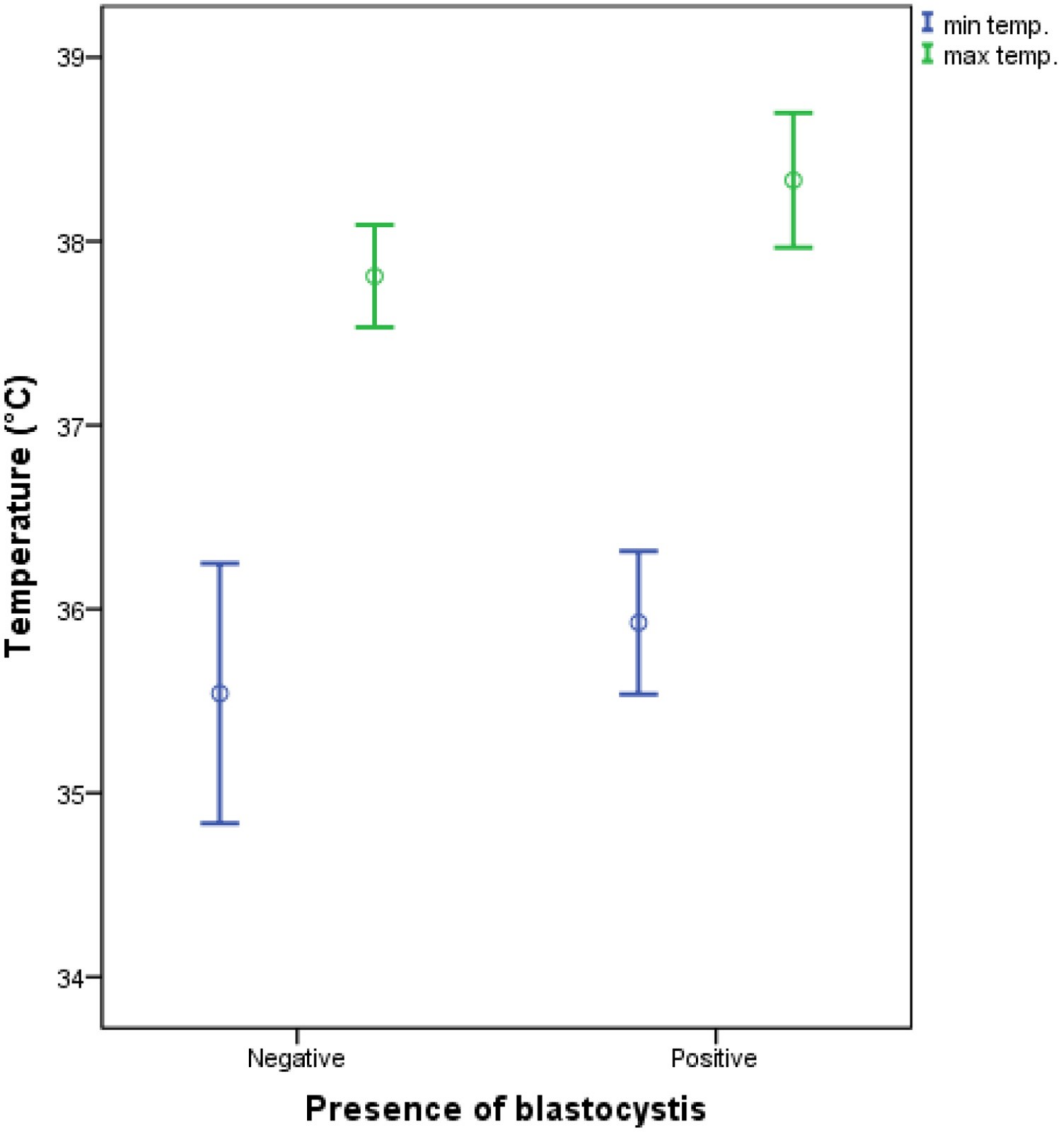
Error bar chart of the minimum (min) and maximum (max) temperature (temp.) reached by *Blastocystis* sp. non-infected (negative) and infected (positive) dengue patients.

It was interesting to show that dengue patients who were positive for *Blastocystis* sp. infection denoted a significantly higher fever rate reaching 38.73°C (p<0.05) compared to the non-*Blastocystis* infected patients (38.44°C) (Table 2). High fever also coupled together with a significantly low platelet count (36.62) for *Blastocystis* sp. infected dengue patients (p<0.05). The duration of admission of *Blastocystis* sp. infected dengue patients were also found to be significantly longer (4.33 days) (p<0.05) compared to the non-*Blastocystis* sp. infected dengue patients (3.71 days). This showed that there could be an association between high body temperature, low platelet count, frequency of going to toilet, and duration of admission period of a patient having both dengue and *Blastocystis* sp. infection. The error bar chart (Fig 1) shows a clearer range of the maximum body temperature reached by the *Blastocystis* sp. infected (positive) and non-infected groups (negative).

**Table 2:** Admission period and body temperature range of *Blastocystis* sp. infected and non-infected dengue patients.

Parameters	*Blastocystis* sp. Positive		*Blastocystis* sp. Negative		p value
	Mean	SD	Mean	SD	
Admission period (n = 89)(days)	4.33	1.278	3.71	0.978	0·019*
Maximum temperature (n = 37) (°C)	38.73	0·3201	38.44	0.5407	0.045*
Minimum platelet count (n = 37)	36.62	27.01	67.83	47.89	0.038*

* Represents significance at p< 0.05

**Fig 2:**
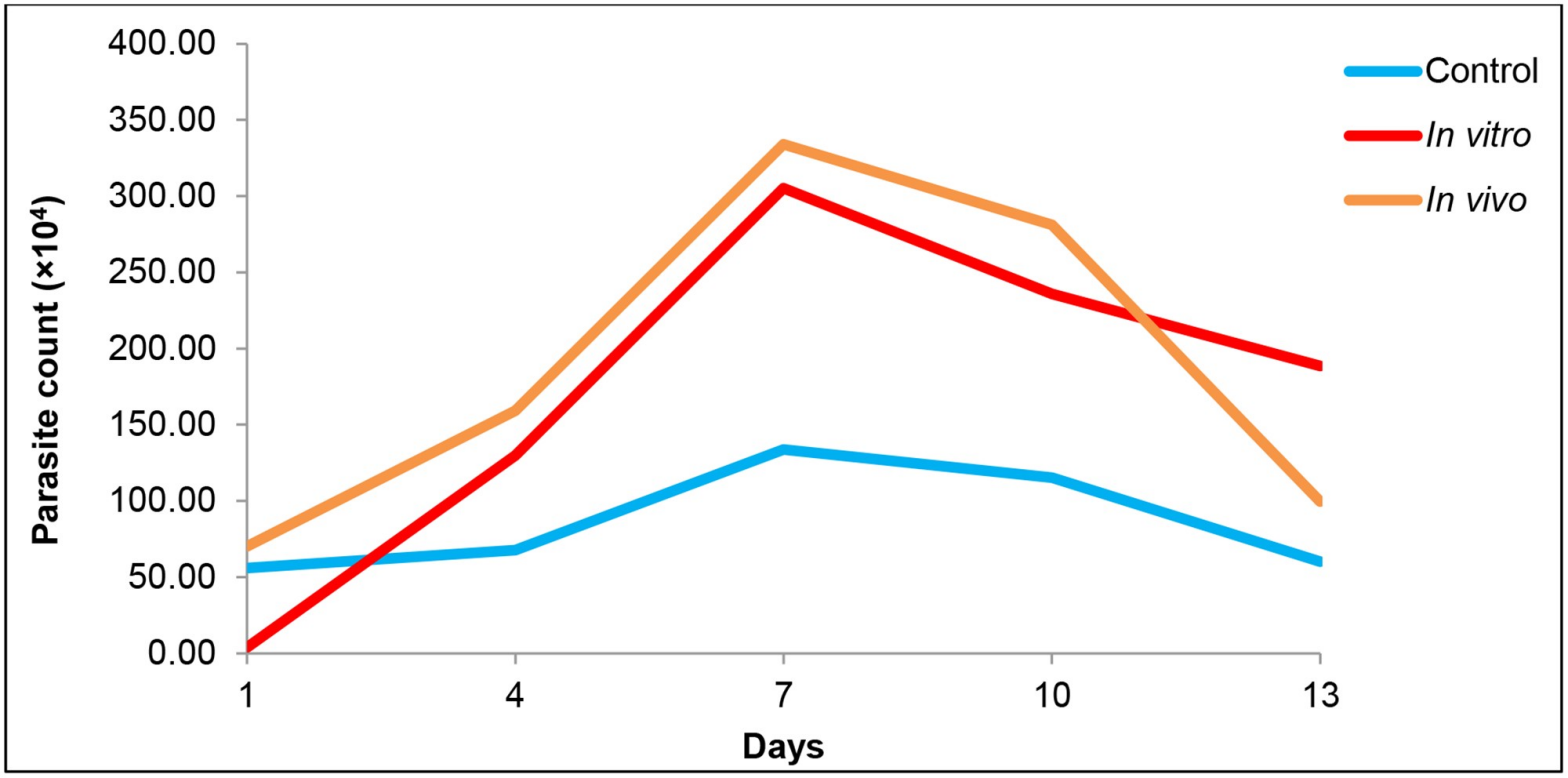
Growth pattern of *Blastocystis* sp. at various thermal conditions. Non-dengue parasite cultures were incubated throughout at 37°C (control), and at 41°C for 24 hours and re-cultured at 37°C (in vitro thermal stress). Parasite isolated from dengue patients were classified as the third group (in vivo thermal stress). Isolates obtained from dengue patients (*in vivo*) showed the highest growth compared to the other groups.

Growth profile analysis of the three different thermal groups showed peak growth on day 7. On day 7, both the *in vitro* and *in vivo* thermal stressed *Blastocystis* sp. groups showed significantly higher growth (p<0.001) reaching 305.30×10^4^ and 334.00×10^4^ cells/ml respectively compared to the *in vitro* control, 133.30×10^4^ cells/ml (Fig 2). However, the *in vivo* group showed slightly higher growth throughout ten days compared to *in vitro* thermal stressed group. This shows *Blastocystis* sp. could multiply at a higher rate upon exposure to high temperature *in vitro* and *in vivo*.

## Discussion

*Blastocystis* sp. in *in vitro* cultures showed a higher growth rate with a corresponding increase in temperature [10]. This concurs with our current observation where *Blastocystis* sp. isolated from dengue patients showed a higher growth compared to the parasites maintained in *in vitro* cultures at 37°C. Dengue patients infected with *Blastocystis* sp. also showed a significantly higher temperature compared to the non-*Blastocystis* sp. infected patients. This can be explained by the role of pro inflammatory cytokines in immune regulation during infection. Upon exposure to viral infection (i.e. dengue virus), white blood cells secrete chemicals known as pyrogens. These pyrogens by flowing in the bloodstream make their way to the hypothalamus, which in charge of regulating body temperature. When pyrogens bind to certain receptors in the hypothalamus, body temperature rises in the attempt of killing the invading pathogen. Some common pyrogens are known as Interleukin- (IL-) 1, 6, and tumor necrosis factor alpha (TNF-α). Moreover, various studies have reported that *Blastocystis* sp. enhance the production of pyrogens in various circumstances. Our previous study showed that IL-6 and IL-8 secreted by *Blastocystis* sp. antigen enhanced the cancer cell growth [24,25] which concurred with another study in Singapore where it was revealed that *Blastocystis* sp. antigens stimulate the production of a variety of cytokines, such as IL-1β, IL-6, and TNF-α [26]. Apart from that, these pro-inflammatory cytokines were also found higher in the plasma and peripheral blood mononuclear cells of IBS patients [27,28]. These findings imply that the presence of *Blastocystis* sp. during dengue infection triggers the increase of temperature which could be due to highly elevated pro inflammatory cytokines by both parasitic and virus infection. This could justify why the temperature in *Blastocystis* sp. infected dengue patients is higher compared to the non-infected ones which triggers a greater parasite multiplication rate and causing the aggravation of the gastrointestinal symptoms.

Gastrointestinal symptoms and signs are very common among dengue patients [29–31]. This has also been reported among dengue patients in Malaysia [11,32]. Dengue patients presenting with vomiting, nausea, abdominal pain, abdominal tenderness, and diarrhea have been suggested to be given closer observation as intensive care is required [32]. The suggestion that gastrointestinal symptoms and signs have a relationship with presence of dengue, clearly underlines the importance of identifying the real source of these symptoms lest they are mistaken to be caused by the dengue virus [31]. Our current study suggests that, gastrointestinal symptoms among dengue patients could have been contributed by *Blastocystis* sp. This could justify an increase in the period of hospitalization in such *Blastocystis* sp. infected dengue patients.

Dengue has been identified by Ministry of Health, as the ninth rank causing level of burden as diarrheal diseases in Malaysia [33]. The study has also revealed the costing for hospital services for dengue patients in Malaysia as an average of RM771 (USD184.05) and RM877 (USD209.36) per bed-day for public and private hospitals respectively [33]. Based on our observation, we have identified that *Blastocystis* sp. infected dengue patients tend to be hospitalized significantly longer (4.33 days) compared to the non-*Blastocystis* sp. infected patients (3.71 days). Thus, at a public hospital, treatment cost for a *Blastocystis* sp. infected dengue patient is an average of RM3338.43 (USD796.95), while for the non-*Blastocystis* sp. infected dengue patient is RM2860.41 (USD682.84). This shows that, a *Blastocystis* sp. infected dengue patient could cause an extra cost of RM478 (USD114.11) to the public hospital. Even though it is just a slight difference, in long run this scenario has the probability of increasing the hospital services costing which could accommodate the treatment of more dengue patients.

Normally, stool examination is not routinely carried out for all dengue patients unless they are presented with severe gastrointestinal symptoms. Often prior to acquiring dengue, *Blastocystis* sp. infected patients may remain asymptomatic and therefore do not resort to treatment [34]. Therefore, the pathogenicity of *Blastocystis* sp. appears to be influenced by numbers as suggested by Nimri (1993), who stated that parasite more than five per field at a magnification of ×400 does pose a problem [35]. Since, the thermal stressed *Blastocystis* sp. induces higher proliferation, this could exacerbate the gastrointestinal symptoms among *Blastocystis* sp. infected dengue patients. Hence, dengue patients with gastrointestinal symptoms should be screened for *Blastocystis* sp. to enable earlier detection and hospital discharge reducing the higher demands for beds especially in limited health care settings located in dengue endemic areas.

We agree that our study have limitations such as the recruitment of a larger number of dengue patients. Even though approximately more than 300 patients were met individually, written consent was obtained and interviewed, but only 25% were responded by providing their stool sample. This was mainly because most patients were not warded in the same ward all the time and their stool cups were misplaced during room shifting, thus we lost many stool samples during this process. Some patients were even discharged sooner before they could provide us their stool samples and there were also patients who said that they were not comfortable collecting fecal samples in hospital toilets. This has also limit us from collecting stool sample at different time point of illness to find the direct correlation with temperature. However, the study has provided evidence which can be an important contribution to public health.

## Conclusion

As this study is the first to assess the prevalence of *Blastocystis* sp. infection among dengue patients, further research need to be carried out to determine the direct correlation between *Blastocystis* sp. and dengue infection. Firstly, the severity of dengue symptoms should be correlated with presence of *Blastocystis* sp. infection. Second, it is also important to make correlation between different age groups, as this can influence the susceptibility towards dengue and *Blastocystis sp*. infection in both adult and pediatric population. Lastly, dengue patients positive with *Blastocystis* sp. infection should be treated to assess whether the gastrointestinal symptoms especially severity of diarrhea and other symptoms can be reduced or eliminated.
